# Telmisartan attenuates kidney apoptosis and autophagy-related protein expression levels in an intermittent hypoxia mouse model

**DOI:** 10.1007/s11325-018-1720-9

**Published:** 2018-09-15

**Authors:** Xiao-Bin Zhang, Jing-Huang Cai, Yu-Yun Yang, Yi-Ming Zeng, Hui-Qing Zeng, Miao Wang, Xiao Cheng, Xiongbiao Luo, Henry Chidozie Ewurum

**Affiliations:** 1 Department of Respiratory Medicine, Zhongshan Hospital, Xiamen University, No.201, Hubin Nan Road, Siming District, Xiamen, 361004 Fujian Province People’s Republic of China; 2 Teaching Hospital of Fujian Medical University, Xiamen, China; 3 Department of Pulmonary and Critical Care Medicine, The Second Affiliated Hospital of Fujian Medical University, No. 34, Zhongshanbei Road, Licheng District, Quanzhou, 362000 Fujian Province China; 4 The Second Clinical Medical College of Fujian Medical University, Quanzhou, China; 5 Center of Respiratory Medicine of Fujian Province, Quanzhou, China; 6 Department of Computer Science, Xiamen University, Xiamen, Fujian China

**Keywords:** Telmisartan, Intermittent hypoxia, Renal, Apoptosis, Autophagy

## Abstract

**Purpose:**

Obstructive sleep apnea (OSA) is associated with renal impairs. As a novel pathophysiological hallmark of OSA, chronic intermittent hypoxia (CIH) enhances apoptosis and autophagy. The present study aims to evaluate the effect of telmisartan on CIH-induced kidney apoptosis and autophagy in a mouse model of OSA.

**Materials and methods:**

Mice were randomly allocated to normoxia, CIH, and CIH+telmisartan groups (*n* = 12 in each group). The CIH exposure duration was 12 weeks. Mice in the CIH+telmisartan group received telmisartan administration. The terminal deoxynucleotidyl transferase dUTP nick-end labeling (TUNEL) assay and western blotting of Bax and cleaved caspase-3 were conducted for evaluating apoptosis in kidney tissue. While the autophagy-related proteins, beclin-1 and LC3, were also observed via western blotting.

**Results:**

The percentage of apoptotic cell in the CIH group was significantly higher than that of normoxia group; meanwhile, Bax and cleaved caspase-3 protein levels were increased in the CIH group than those of normoxia group (all *p* < 0.05). Compared with the normoxia group, mice in the CIH group had greater autophagy-related proteins (beclin-1 and LC3) expression. When compared to the CIH group, both the renal apoptosis and autophagy in the CIH+telmisartan group were decreased.

**Conclusion:**

The CIH accelerates renal apoptosis and autophagy levels. Telmisartan ameliorating those levels suggests that it might prevent renal impairs from the CIH in OSA patients.

## Introduction

Obstructive sleep apnea (OSA) is a highly prevalent medical disorder among middle-age adults [1]. Accumulated data confirmed the bidirectional association between kidney diseases and OSA; for one thing, the incidence and mortality of OSA in kidney disease are higher than those in general population; for the other, OSA contributes the impairs of renal function [2, 3]. Our previous studies indicated that cystatin C, a biomarker of early renal impairs, was higher in several OSA patients without complications [4], and continuous positive airway pressure (CPAP) treatment can normalize cystatin C levels in those patients [5]. The potential mechanisms of OSA-related renal impair are inconclusive. Previous studies elucidated that OSA leads to renal impairs thorough hypertension, sympathetic nervous system and renin-angiotensin-aldosterone system overactivation, endothelial dysfunction, and increased oxidative stress [6, 7].

Apoptosis and autophagy are two important cellular processes with complex and intersecting networks. Evidence shows that hypoxia is closely related with both apoptosis and autophagy [8, 9]. Chronic intermittent hypoxia (CIH) is a novel pathophysiological hallmark of OSA [1]. A study by Liu and colleagues [10] concluded that CIH induces differential expression of miRNAs which was associated with apoptosis or autophagy-related gene expression. Previous studies also showed that CIH enhances both hippocampal and myocardial apoptosis in rat mimicking OSA, and telmisartan administration can relieve the apoptosis levels [11, 12]. Autophagy in hippocampal neurons can be also aggravated by intermittent hypoxia (IH) [13]. However, data addressing the effect of CIH on apoptosis and autophagy levels in the kidney are scarce.

The present study aims to evaluate the effect of CIH on renal apoptosis and autophagy and further to assess the influence of telmisartan on those in a mouse model.

## Materials and method

### Animals and subgroups

Thirty-six 7-week-old male C57BL/6 mice were purchased from Chinese Academy of Science Laboratory Animals Center in Shanghai, China. Mice were kept in a departmental animal facility on a 12:12-hour light-dark cycle, and free access to water and food, and were randomly assigned to the following groups (*n* = 12 in each group): normoxia, chronic intermittent hypoxia (CIH), and intermittent hypoxia plus telmisartan (CIH+temisartan). The body weights of mice in each group were measured every week. This protocol was approved by the ethics committee in Zhongshan Hospital, Xiamen University, and conducted in accordance with the Guide for the Care and Use of Laboratory Animals [14].

### Chronic intermittent hypoxia exposure

The protocol of chronic intermittent hypoxia exposure was based on previous studies [15, 16]. Briefly, mice in the CIH and CIH+temisartan groups (*n* = 24) were placed in a plexiglass chamber with one-way valves, and a programmable instrument which regulated the flow of oxygen, nitrogen, and compressed air into the chamber. This system ensured the oxygen concentration in the chamber varying from 21 to nadir 5–7%. The cycle time of hypoxia and reoxygenation is 2 min. The experimental period of CIH exposure was from 08:00 AM to 04:00 PM daily for 12 consecutive weeks.

### Drug administration

Mice in CIH+telmisartan group were administered telmisartan (10 mg/kg dissolved in double-distilled water), while mice in the normoxia and CIH groups were administrated equal volume of double-distilled water. The drug administration by oral gavage was from the third week to the end of the CIH exposure.

### Tissue preparation

After 12 weeks of the experimentation, mice were anesthetized with pentobarbital and exsanguinated by cardiac puncture. Blood samples were collected and centrifuged at 3000*g* for 15 min at 4 °C; then, the supernatants (serum) were collected and stored at − 80 °C for future analysis. Kidneys were either frozen in liquid nitrogen then transferred to − 80 °C refrigerator for further analysis or fixed in buffered 10% formalin for histological examination and immunohistochemistry. Kidney tissues were homogenized by RIPA lysis buffer (Beyotime, Beijing, China), then centrifuged at − 4 °C. After the supernatants were extracted, the protein concentrations were detected with bicinchoninic acid protein assay (Beyotime, Beijing, China); then, the supernatants were preserved in − 80 °C refrigerator.

### Detection of blood urea nitrogen, serum creatinine, and serum and kidney angiotensin II concentration

Blood urea nitrogen and serum creatinine were analyzed using a Hitachi 7020 automatic analyzer (Hitachi Co. Ltd., Tokyo, Japan). The levels of angiotensin II in serum and kidney homogenates were determined by enzyme-linked immunosorbent assay (ELISA) using mouse angiotensin II immunoassay kit according to the instruction of the manufacturer (RayBiotech, Inc., USA).

### Hematoxylin and eosin staining and TUNEL assay

Kidney tissue of each group in the formalin was further embedded in paraffin and sectioned in 5-μm slices. Slices were stained with hematoxylin and eosin (HE). A commercially available terminal deoxynucleotidyl transferase dUTP nick-end labeling (TUNEL) staining kit (Roch, China) was used to detect apoptosis according to the manufacturer’s instructions. The numbers of apoptotic cells and total cell in the visual field (× 400 magnification) were determined. The results are presented as the percentage of apoptotic cells among the total cell population. Leica DM2500 microscope was used for photographing.

### Western blotting

The supernatants of kidney tissue were used for analyzing the expression of special proteins. The protein expressive levels of angiotensin II receptor type 1 (AGTR1), Bax, caspase-3, autophagy-related protein LC3, and beclin-1 were evaluated by western blotting. Equal protein (40 μg/band) from the supernatants of each group were subjected to 12% SDS-PAGE and transferred to PVDF membranes. Membranes were blocked in 5% non-fat dry milk in TBST (10 Mm Tris-HCL, pH 7.5, 150 Mm NaCL, 0.05% Tween-20) for 1 hour at room temperature. After washing by TBST for three times, membranes were incubated with rabbit-anti-AGTR1 (Novus Biologicals, Littleton, CO, USA), rabbit-anti-Bax (Cell Signaling Technology, Beverly, MA, USA), rabbit anti-caspase-3 (Cell Signaling Technology, Beverly, MA, USA), rabbit anti-beclin-3 (Cell Signaling Technology, Beverly, MA, USA), rabbit anti-LC3 (Novus Biologicals, Littleton, CO, USA), and mouse anti-β-actin (Santa Cruz Biotechnology, USA) overnight at 4 °C. After incubated with a secondary antibody conjugated to horseradish peroxidase at room temperature for 1 hour, membranes were developed and exposed using an enhanced chemiluminescence kit (Clarity™ Western ECL Substrate, Bio-Rad). The band densities on the membranes were estimated using the ImageJ analysis software (National Institutes of Health, Bethesda, MD, USA). All experiments were repeated triplicate.

### Statistical analysis

GraphPad Prism software 5.0 (GraphPad Software, Inc., La Jolla, CA, USA) was used to assess the data. All data are shown as a mean ± standard deviation and compared using one-way analysis of variance and further post hoc test for further comparison between groups. A *p* value less than 0.05 was considered as statistical significance.

## Results

### Change in body weight, blood urea nitrogen, serum creatinine, and angiotensin II in each group

**Fig. 1:**
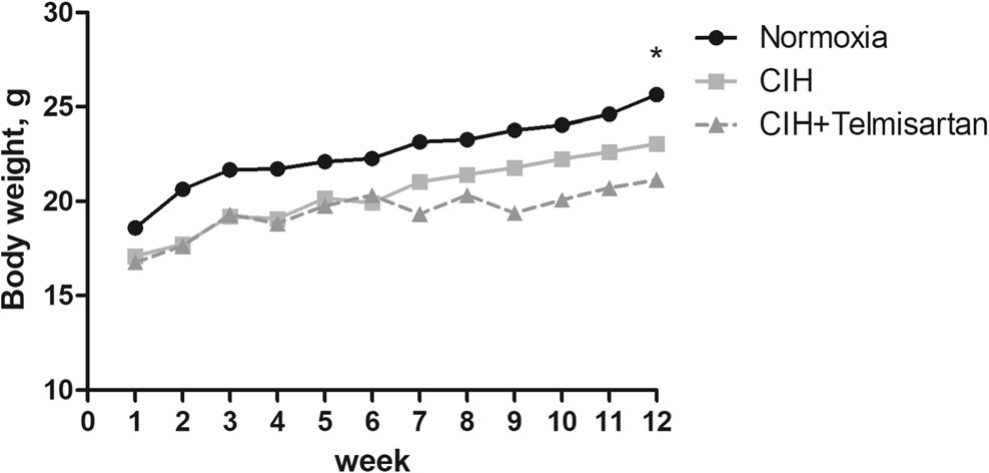
Body weight in each group. Mice in the normoxia group were significantly heavier than those in the CIH and CIH+telmisartan groups at the twelfth week of the experimental period. *p < 0.001 when compared with the normoxia group. CIH chronic intermittent hypoxia

**Fig. 2:**
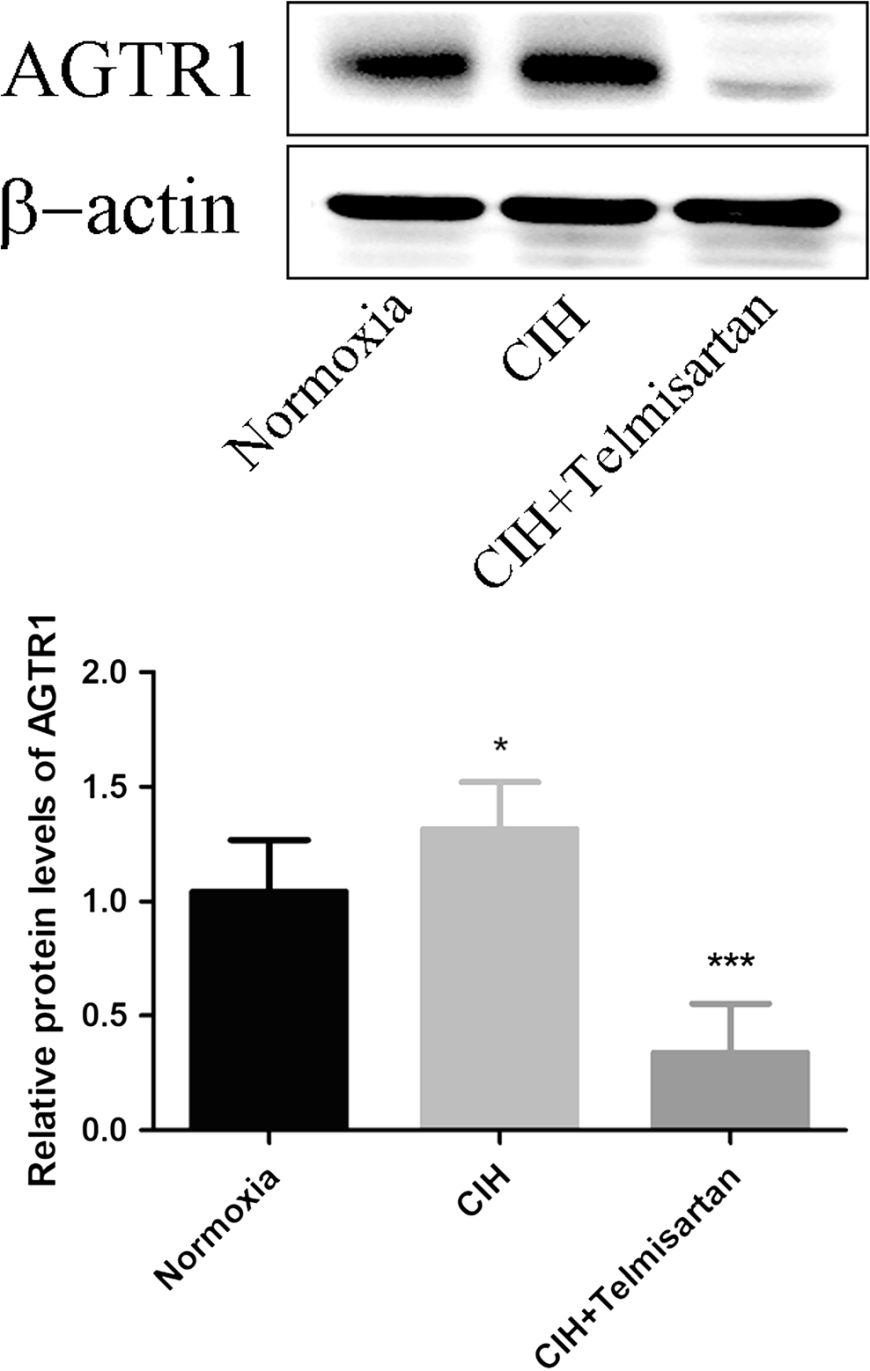
Western blotting for AGTR1 protein expression. The AGTR1 protein levels in the CIH group significantly increased when compared to the normoxia group (*p* < 0.05). Treatment with telmisartan obviously decreased the AGTR1 expression in kidney tissue (compared to the CIH group, *p* < 0.001). AGTR1 angiotensin II receptor type 1, CIH chronic intermittent hypoxia

Mice in normoxia group (25.64 ± 1.59 g) gained more body weight than those in the CIH (23.05 ± 0.95 g) and CIH+telmisartan (21.15 ± 1.15 g) groups at the twelfth week of experimentation (*p* < 0.001) (Fig. 1). No difference in body weight between the CIH and CIH+telmisartan groups (*p* > 0.05) (Table 1). No significant differences in blood urea nitrogen and serum creatinine between groups. When compared with the normoxia and CIH groups, increased serum and kidney tissue angiotensin II levels were detected in the CIH+telmisartan group (*p* < 0.001) (Table 1). Compared with the normoxia group, the expression of AGTR1 was higher in the CIH group. After telmisartan administration, its expression decreased (Fig. 2).

**Table 1:** Effects of telmisartan treatment on blood urea nitrogen (BUN), serum creatinine, and serum and kidney tissue angiotensin II levels in chronic intermittent hypoxia-induced mice

	Normoxia	CIH	CIH+telmisartan
BUN (mmol/L)	11.63 ± 2.90	11.54 ± 0.85	11.60 ± 0.99
Serum creatinine (μmol/L)	12.67 ± 1.43	11.88 ± 0.68	12.13 ± 1.02
Serum angiotensin II (pg/ml)	229.54 ± 20.98	241.69 ± 23.49	302.46 ± 19.46^*^
Kidney tissue angiotensin II (pg/100 mg tissue)	247.43 ± 45.59	216.99 ± 13.65	306.50 ± 20.97^*^

*p < 0.001 when compared with the normoxia and CIH groups

*BUN* blood urea nitrogen, *CIH* chronic intermittent hypoxia

### Kidney histopathological changes

**Fig. 3:**
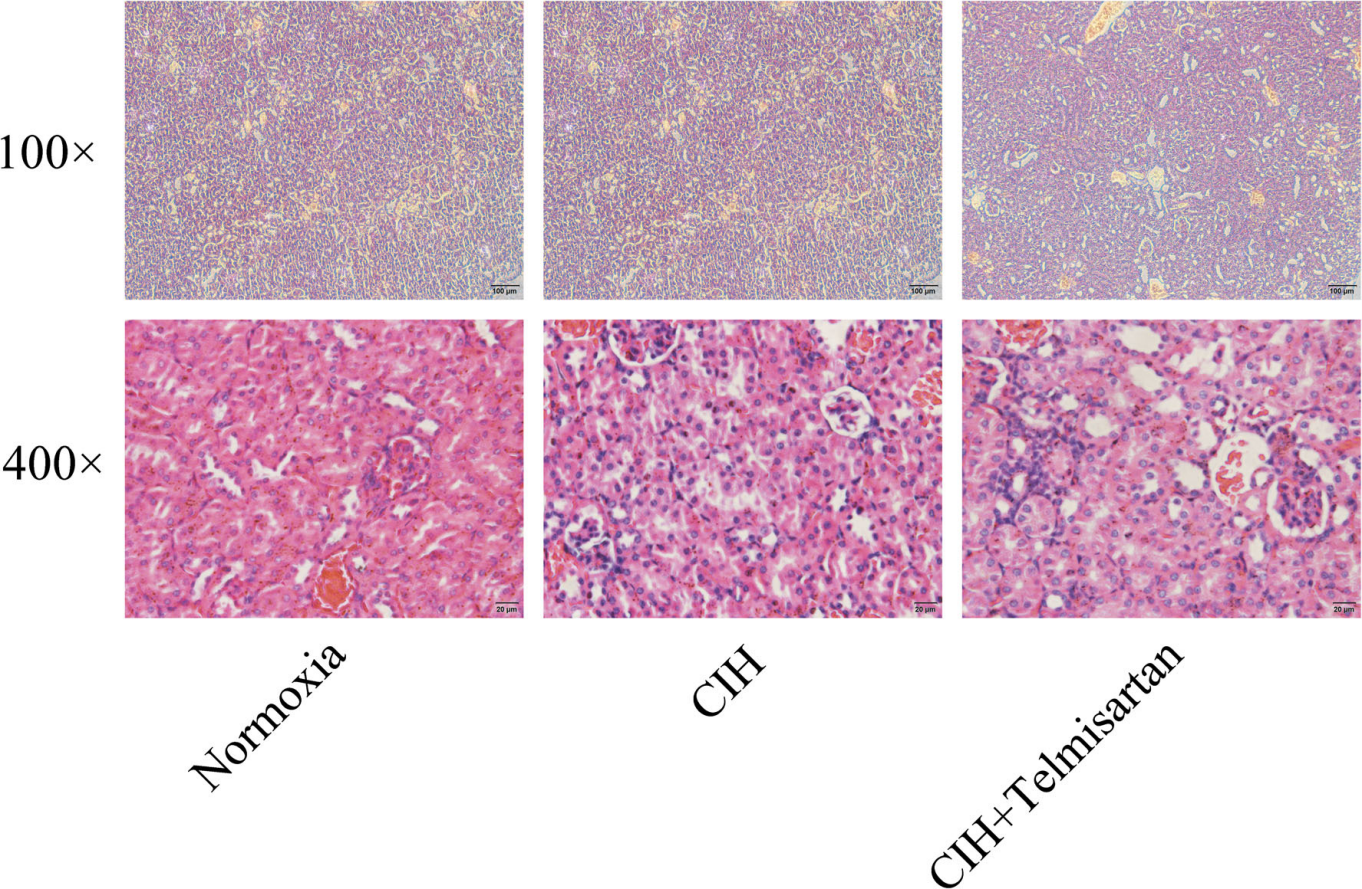
Kidney histopathological changes. The HE staining results illustrated that no abnormal architecture was found in all groups. CIH chronic intermittent hypoxia, HE hematoxylin and eosin staining

To investigate whether CIH and telmisartan influence the kidney architecture, a histopathological analysis of kidney tissue stained by hematoxylin and eosin were performed. After reviewing × 100 and × 400 magnified images, no abnormal architecture of the glomerular and tubular was detected in all groups (Fig. 3).

### Effect of telmisartan on apoptosis in mice subjected to the CIH

**Fig. 4:**
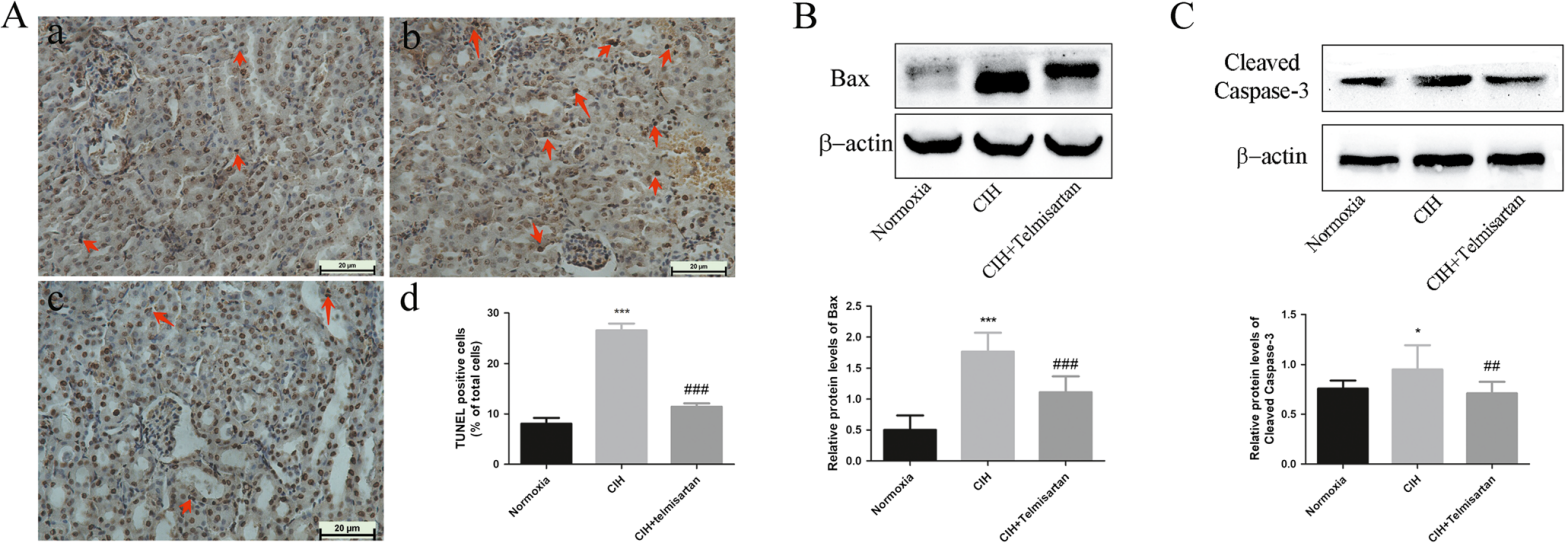
Effect of telmisartan on apoptosis induced by CIH. The percentage of apoptotic cells (mostly tubular cells) was significantly higher in the CIH group than that of the normoxia group. After treating with telmisartan, the apoptotic percentage in the CIH+telmisartan group was lower than that of the CIH group (A). Mice in the CIH group had higher Bax (B) and cleaved caspase-3 (C) protein levels than those in the normoxia group. In comparison to the CIH group, these proteins levels were decreased in the CIH+telmisartan group (B, C). ***p < 0.001 when compared with normoxia group; *p < 0.05 when compared with normoxia group; ###*p* < 0.001 when compared with the CIH group; ##*p* < 0.01 when compared with the CIH group. CIH chronic intermittent hypoxia

TUNEL staining results depicted that the percentage of apoptotic cells, mostly tubular cells, in the CIH group was significantly higher than that of the normoxia group. After treatment with telmisartan, the apoptosis rate was decreased significantly (Fig. 4A). Both Bax and cleaved caspase-3 were increased in the CIH group than in normoxia group, while these protein levels were decreased in mice receiving telmisartan treatment (Fig. 4B, C).

### Effect of telmisartan on autophagy in mice subjected to the CIH

**Fig. 5:**
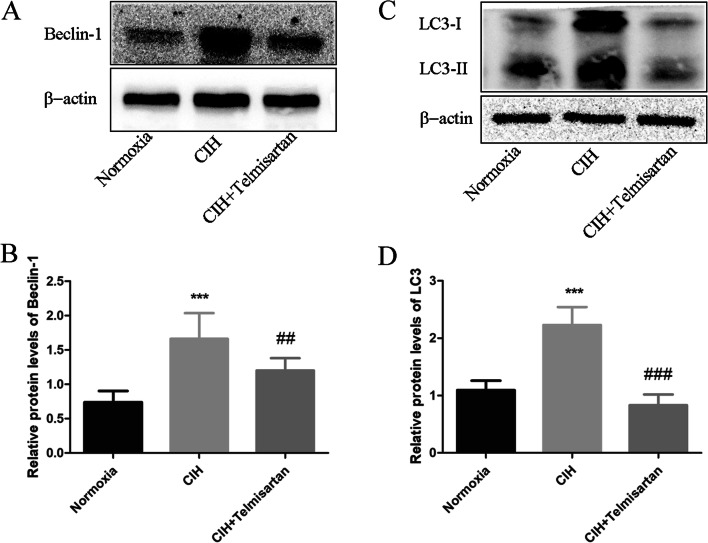
Effect of telmisartan on autophagy induced by CIH. Western blotting results showed that the beclin-1 levels were higher in the CIH group than those of normoxia group, while the levels were decreased after mice received telmisartan treatment (Fig. 5 A, B); CIH induced high expression of LC3, while telmisartan can attenuate LC3 expression (Fig. 5 C, D). ***p < 0.001 when compared with normoxia group; ##*p* < 0.01 when compared with the CIH group; ###*p* < 0.001 when compared with the CIH group. CIH chronic intermittent hypoxia

Compared to mice exposed to normoxia, the expressions of autophagy-related proteins, beclin-1 and LC3, were significantly increased in mice subjected to the CIH. After receiving telmisartan treatment, these protein levels were decreased (Fig. 5).

## Discussion

The present study demonstrated that CIH accelerates the progression of apoptosis and autophagy in kidney tissue of a mouse model mimicking OSA. We further found that telmisartan attenuates the CIH-induced apoptosis and autophagy.

The relationship between sleep apnea and kidney impair/disease is confirmed by several previous studies [3–5, 17–20]. A population-based cohort study in Taiwan demonstrated that patients with sleep apnea had a 1.94-fold and 2.2-fold increase in the incidences of chronic kidney disease and end-stage renal disease [18]. Our previous study showed that cystatin C, a novel biomarker of kidney impair, was increased in patients with an apnea-hypopnea index of more than 30 events/hour [4]. A systematic review and meta-analysis concluded the significant association between OSA and higher albuminuria/proteinuria and a lower estimated glomerular filtration rate [19]. A study indicates that CPAP treatment can improve renal hemodynamics and the downregulation of the renal renin-angiotensin system in OSA patients [21]. Previous experimental studies indicated that the potential mechanisms of OSA induced renal impairs include overactivation of the sympathetic nervous system and renal-angiotensin system, high oxidative stress and inflammation levels, endothelial dysfunction, and high glomerular perfusion [20, 22–24]. Intermittent hypoxia also contributes to histological kidney damage and high growth factor expression in an mouse model mimicking OSA [25].

Both apoptosis and autophagy are common in subjects with renal injury [26–29]. Hypoxia causes renal injury partly through apoptosis and autophagy [26, 30]. A study by Wu and co-authors found that CIH results in tubular endothelial apoptosis, and soluble receptor for advanced glycation end products can ameliorate the apoptosis levels. As far as author’s knowledge, few studies focus on the renal apoptosis and autophagy levels in the CIH or OSA subjects. This study demonstrated that CIH induces renal apoptosis and autophagy in a mouse model of OSA. It is postulated that the high CIH-induced apoptosis and autophagy levels are a pathogenesis of OSA enhancing the progression of renal impairs.

As an angiotensin II type 1 receptor blocker (ARB), telmisartan is a widely useful anti-hypertensive drug. Accumulated evidence proved that ARB attenuates CIH-related organ damage [11, 12, 31–36]. An experimental study from our group illustrated that telmisartan protects cardiocytes against CIH-induced oxidative stress [31]. The previous study showed that pre-CIH telmisartan administration ameliorates myocardial injury by attenuating CIH-induced myocardial apoptosis [12]. Meanwhile, the CIH-induced hippocampal apoptosis also can be attenuated by telmisartan via regulating nitric oxide synthase activity, inhibiting nitric oxide product, and decreasing lipid peroxidation and inflammatory response [11]. The present study with a mouse model of the CIH (12-week exposure of intermittent hypoxia) found that the renin-angiotensin-aldosterone system (RAAS) was blocked by telmisartan as previous studies showed [37, 38]. Furthermore, we proved that telmisartan administration ameliorates both apoptosis and autophagy levels in renal tissue. We speculated that telmisartan has the renal protective function in OSA patients, and it is suitable for OSA patients with hypertension or cardiovascular diseases.

Several limitations of this study should be noted. Firstly, normoxia+telmisartan group should have been appropriate, since apoptosis and autophagy levels could be also affected by telmisartan administration in normoxia condition. Secondly, only HE staining was conducted to evaluate the effect of the CIH and telmisartan on kidney architecture change, transmission electron microscopy should be performed to observe the ultrastructural change of kidney in a future study. Thirdly, similar to previous several studies [11, 12, 31, 37], omission of blood pressure measurement was a major limitation of our study. We postulated that not only improving the apoptosis and autophagy levels but also a possible lowering of blood pressure could telmisartan relieve CIH-induced kidney damage. Further experimental study is expected to classify the relationship between CIH, blood pressure change, and kidney damage. Finally, the potential biomechanisms which CIH leads to renal tubular apoptosis and autophagy and the function of telmisartan were not under discussion in the present study.

## Conclusions

The present study elucidated that both apoptosis and autophagy levels were increased in kidney tissue of a CIH mouse model. Telmisartan ameliorates both levels. It is postulated that telmisartan can be considered as a potential drug for reliving the renal impairs in OSA patients thorough attenuating renal apoptosis and autophagy levels.
